# Chronic Glycaemic Control Modulates the Relationship Between GIP and Glucagon Secretion Following Oral and Enteral Nutrients in Type 2 Diabetes

**DOI:** 10.1111/dom.70735

**Published:** 2026-04-07

**Authors:** Zhenxi Wang, Weikun Huang, Rasmus S. Rasmussen, Michael Horowitz, Karen L. Jones, Christopher K. Rayner, Cong Xie, Tongzhi Wu

**Affiliations:** ^1^ School of Medicine, College of Health Adelaide University Adelaide Australia; ^2^ Centre of Research in Translating Nutritional Science to Good Health Adelaide University Adelaide Australia; ^3^ College of Medicine and Public Health Flinders University Adelaide Australia; ^4^ Faculty of Health and Medical Sciences, Department of Biomedical Sciences University of Copenhagen Copenhagen Denmark; ^5^ Endocrine and Metabolic Unit, Royal Adelaide Hospital Adelaide Australia; ^6^ Department of Gastroenterology and Hepatology Royal Adelaide Hospital Adelaide Australia

**Keywords:** GIP, glucagon, glycaemic control, incretin physiology, type 2 diabetes

## Abstract

**Aims:**

Glucose‐dependent insulinotropic polypeptide (GIP) may exert both insulinotropic and glucagonotropic effects. While its insulinotropic action is reportedly attenuated or abolished with worsening of glycaemic control in type 2 diabetes (T2D), the relationship between GIP and glucagon secretion across different levels of glycaemic control remains unclear. This study evaluated the relationship between postprandial GIP and glucagon responses to a mixed meal in T2D and to intraduodenal fat and glucose infusion in healthy and/or T2D individuals.

**Materials and Methods:**

Data were analysed from three clinical studies: mixed‐meal testing in T2D (*n* = 79), intraduodenal fat infusion (2 kcal/min over 120 min) in T2D (*n* = 15) and intraduodenal glucose infusion (2 kcal/min over 60 min) in health and T2D (*n* = 10 each). Relationships between postprandial GIP and glucagon or insulin following the mixed meal were also determined after stratification by HbA1c (< 6.5%, 6.5%–7.0%, > 7.0%).

**Results:**

Following mixed‐meal ingestion, early postprandial (0–30 min) glucagon secretion correlated positively with GIP, with the strength of this association increasing with higher HbA1c levels. In contrast, the insulinogenic index correlated with GIP only in subgroups with HbA1c < 6.5% and between 6.5%–7.0%. During intraduodenal fat infusion, glucagon and GIP responses correlated strongly in T2D. Following intraduodenal glucose infusion, early glucagon and GIP increments (0–15 min) were closely related in T2D, but not in healthy individuals.

**Conclusions:**

These observations indicate a glycaemia‐dependent shift in endogenous GIP action in T2D, characterised by attenuation of insulinotropic effects and relative amplification of glucagonotropic effects with deteriorating glycaemic control.

**Trial Registration:** ACTRN12614001131640, ACTRN12614001117606 and ACTRN12615001240538

## 1 Introduction

Glucose‐dependent insulinotropic polypeptide (GIP) and glucagon‐like peptide‐1 (GLP‐1) are well‐established incretin hormones, accounting for ~40%–70% of postprandial insulin secretion in health, that is, the ‘incretin effect’ [1]. They also exhibit divergent effects on pancreatic α‐cells: GLP‐1 suppresses glucagon secretion during hyperglycaemia [2], whereas GIP stimulates glucagon during hypoglycaemia [3]. These complementary actions may contribute to the maintenance of normal glucose homeostasis.

In type 2 diabetes (T2D), the ‘incretin effect’ is attenuated, reflecting, at least partly, a diminished insulinotropic action of GIP [4]. That GLP‐1 retains much of its insulinotropic action in T2D [5] and also suppresses glucagon [1], slows gastric emptying [6] and inhibits energy intake [7], has underpinned the GLP‐1‐based therapies for T2D. However, the recently appreciated substantial metabolic benefits of dual GLP‐1/GIP receptor agonism (e.g., tirzepatide) [8] and GIP receptor antagonism [9] have renewed interest in GIP actions in T2D. While the insulinotropic response to GIP declines with deterioration of chronic glycaemic control [10], its effect on glucagon secretion in T2D (which is often abnormally augmented) is poorly defined. Although exogenous GIP administration has been shown to augment glucagon secretion and exacerbate postprandial glycaemia in suboptimally‐controlled T2D (mean HbA1c ~7.4%) [11], it remains unclear as to whether GIP released endogenously during physiological nutrient stimulation is associated with glucagon secretion and whether this relationship varies according to chronic glycaemic control in T2D. Definition of this relationship has the potential to provide important insights into islet hormone dysregulation in T2D.

Accordingly, the aim of the present study was to examine the relationship between postprandial GIP and glucagon responses following a mixed meal in T2D and to determine whether this association varies with glycaemic control as assessed by HbA1c. Given prior evidence linking postprandial glucagon secretion to gastric emptying [12], we also examined the relationship between the GIP and glucagon responses to intraduodenally infused glucose and fat in healthy and/or T2D individuals.

## 2 Materials and Methods

### 2.1 Participants

Data were derived from three previously reported studies (clinical trial registrations ACTRN12614001131640, ACTRN12614001117606 and ACTRN12615001240538) [2, 13, 14] evaluating the metabolic responses to nutritional or pharmacological therapies in healthy and/or T2D subjects. T2D subjects were managed by diet or metformin monotherapy (ceased 48 h before testing). Exclusion criteria included significant gastrointestinal symptoms or surgery, medications affecting gastrointestinal function or appetite, renal or hepatic impairment and autonomic dysfunction. All studies were approved by the Royal Adelaide Hospital Human Research Ethics Committee, and participants provided written informed consent.

### 2.2 Protocols

All subjects avoided strenuous exercise for 24 h, consumed a standardised beef lasagne meal (2472 kJ, McCain Foods, Melbourne, Vic., Australia) at ~7 PM and then fast overnight before attending our clinical research laboratory. On arrival (~8 AM), an intravenous cannula was inserted into a forearm vein for repeated blood sampling. Specific study procedures are detailed below.

#### 2.2.1 Part 1: Mixed Meal Study in T2D

Seventy‐nine subjects with T2D (Table 1) consumed a semisolid meal (*t* = 0–5 min) consisting of powdered potato (65 g), glucose (20 g), one egg yolk and 13C‐octanoic acid (100 μL) reconstituted with water (200 mL; 368.5 kcal, 61.4 g carbohydrate, 7.4 g protein, 8.9 g fat). Breath samples and venous blood samples were obtained frequently over 240 min for measurements of gastric emptying and blood glucose and plasma glucagon, insulin and total GIP.

**TABLE 1:** Characteristics of participants in three studies. Data are means ± SEM.

	Part 1	Part 2	Part 3	
			Health	T2D
Gender (male/female)	44/35	10/5	10/0	8/2
Age (years)	64.7 ± 0.8	68.8 ± 2.2	45.2 ± 7.8	67.3 ± 2.4
BMI (kg/m^2^)	30.0 ± 0.6	30.2 ± 1.3	25.3 ± 1.3	28.5 ± 1.1
HbA1c (%)	6.6 ± 0.1	6.7 ± 0.2	5.2 ± 0.1	6.2 ± 0.2
Diet/Metformin	40/39	8/7	N/A	6/4
Duration of known diabetes (years)	5.6 ± 0.7	6.6 ± 1.5	N/A	9.3 ± 1.9

#### 2.2.2 Part 2: Intraduodenal Fat Infusion in T2D

Fifteen subjects with T2D (Table 1) were evaluated on a single occasion, when a multi‐lumen silicone catheter (Dentsleeve International, Ontario, Canada) was inserted transnasally, with an infusion port opening 12 cm below the pylorus, as described [2, 14]. A fat emulsion (20% Intralipid; Fresenius Kabi, Hornsby, NSW, Australia) was infused intraduodenally at 2 kcal/min (1 mL/min) from *t* = 0–120 min. Blood samples were collected frequently over 120 min for measurements of plasma glucose, glucagon and total GIP.

#### 2.2.3 Part 3: Intraduodenal Glucose Infusion in Health and T2D

Ten healthy and ten T2D subjects (Table 1) were intubated transnasally with a duodenal infusion port opening 13 cm beyond the pylorus. An aqueous solution containing glucose (30 g) was infused intraduodenally at 2 kcal/min (2 mL/min) during *t* = 0–60 min. Blood samples were collected frequently over 180 min for measurements of blood glucose and plasma glucagon and total GIP.

### 2.3 Measurements

As reported [2, 13, 14], glucose concentrations were measured by glucometer (Optium Xceed; Abbott Laboratories, Lake Bluff, Illinois) in Parts 1 and 3 and by the YSI analyser (2300 STAT Plus; Yellow Springs, Ohio) in Part 2. HbA1c was analysed using high‐performance liquid chromatography (Bio‐Rad Variant II). Plasma insulin was measured by ELISA (catalogue no. 10‐1113; Mercodia, Uppsala, Sweden). Plasma glucagon was measured by radioimmunoassay (RIA) (GL‐32K, Millipore, Billerica, Massachusetts). Due to the documented change in the performance of the Millipore RIA kit following the introduction of a new batch of glucagon antibody in 2019 [15], the glucagon data reported in this study were derived exclusively from the RIA kits produced before 2019. We have validated the performance of these kits against a highly specific sandwich ELISA recognising both the N‐ and C‐terminus of glucagon (Mercodia) [15]. Plasma total GIP was measured by RIA using a modified protocol as described [16]. Gastric emptying was measured using the ^13^C‐octanoic acid breath test, with ^13^CO_2_ in breath samples analysed using non‐dispersive infrared spectrometry (FANci2; Fischer Analysen Instrumente, Leipzig, Germany) and the rate of gastric emptying calculated using the Wagner‐Nelson method, as described previously [17]. Gastric emptying data in Part 1 had been included as a subset in a prior publicaiton [17]. These data were used specifically to examine the relationship between GIP and glucagon responses; the detailed ^13^CO_2_ breath test data are therefore not reproduced here.

### 2.4 Statistical Analysis

Incremental areas under the curve (iAUCs, calculated as the total areas under the curve subtracting the baseline values) for glucose, glucagon, insulin and GIP were calculated over the time interval from baseline to the peak glucagon concentration for each experimental protocol (pre‐defined period). This approach was adopted to capture the physiologically relevant phase of glucagon stimulation, during which endogenous GIP secretion is most likely to influence α‐cell activity, while minimising confounding from later postprandial inhibitory factors (e.g., rising glucose, insulin and GLP‐1). The peak glucagon response occurred at approximately 30 min following the mixed meal, at 120 min during intraduodenal fat infusion (coinciding with the end of the infusion period) and at 15 min during intraduodenal glucose infusion. Accordingly, iAUCs were calculated over 0–30 min, 0–120 min and 0–15 min for the three protocols respectively. The insulinogenic index was calculated as insulin iAUC_0‐30min_ divided by glucose iAUC_0‐30min_. HOMA‐IR was calculated as (fasting glucose × fasting insulin)/22.5. Relationships between the GIP response and the rate of gastric emptying and between the GIP and insulin/glucagon responses were assessed using Pearson correlation analysis after confirming normal distribution of data. In Part 1, subjects were also stratified into three subgroups with HbA1c < 6.5% (*n* = 33), 6.5%–7.0% (*n* = 24) and > 7.0% (*n* = 22) (Table 2). Demographic comparisons between subgroups used one‐way ANOVA, except for sex distribution, which was analysed using either Chi‐square test (Part 1) or Fisher's Exact test (Part 3). All analyses were performed using GraphPad Prism 10.0 (GraphPad Software, San Diego, CA, USA). Data are presented as means ± SEM, with *p* < 0.05 considered statistically significant.

**TABLE 2:** Characteristics of T2D participants stratified by HbA1c (part 1).

	HbA1c < 6.5% (*n* = 33)	HbA1c 6.5%–7.0% (*n* = 24)	HbA1c > 7.0% (*n* = 22)	*p*
Male/Female	20/13	12/12	12/10	0.72
Age (year)	65.3 ± 1.2	65.2 ± 1.5	63.4 ± 1.3	0.54
BMI (kg/m^2^)	29.9 ± 0.9	29.1 ± 1.1	31.1 ± 1.0	0.39
HbA1c (%)	6.3 ± 0.04	6.7 ± 0.1	7.3 ± 0.1	< 0.0001
Diet/Metformin	18/15	14/10	8/14	0.28
HOMA‐IR	2.2 ± 0.3	1.8 ± 0.2	2.4 ± 0.3	0.40

*Note:* Data are means ± SEM. One‐way ANOVA was used to determine statistical differences between the subgroups, except that the distribution of gender and the use of metformin were compared using Chi‐squared analysis.

## 3 Results

### 3.1 Part 1

#### 3.1.1 Gastric Emptying and Glucose, GIP and Glucagon Responses to a Mixed Meal in T2D

**FIGURE 1:**
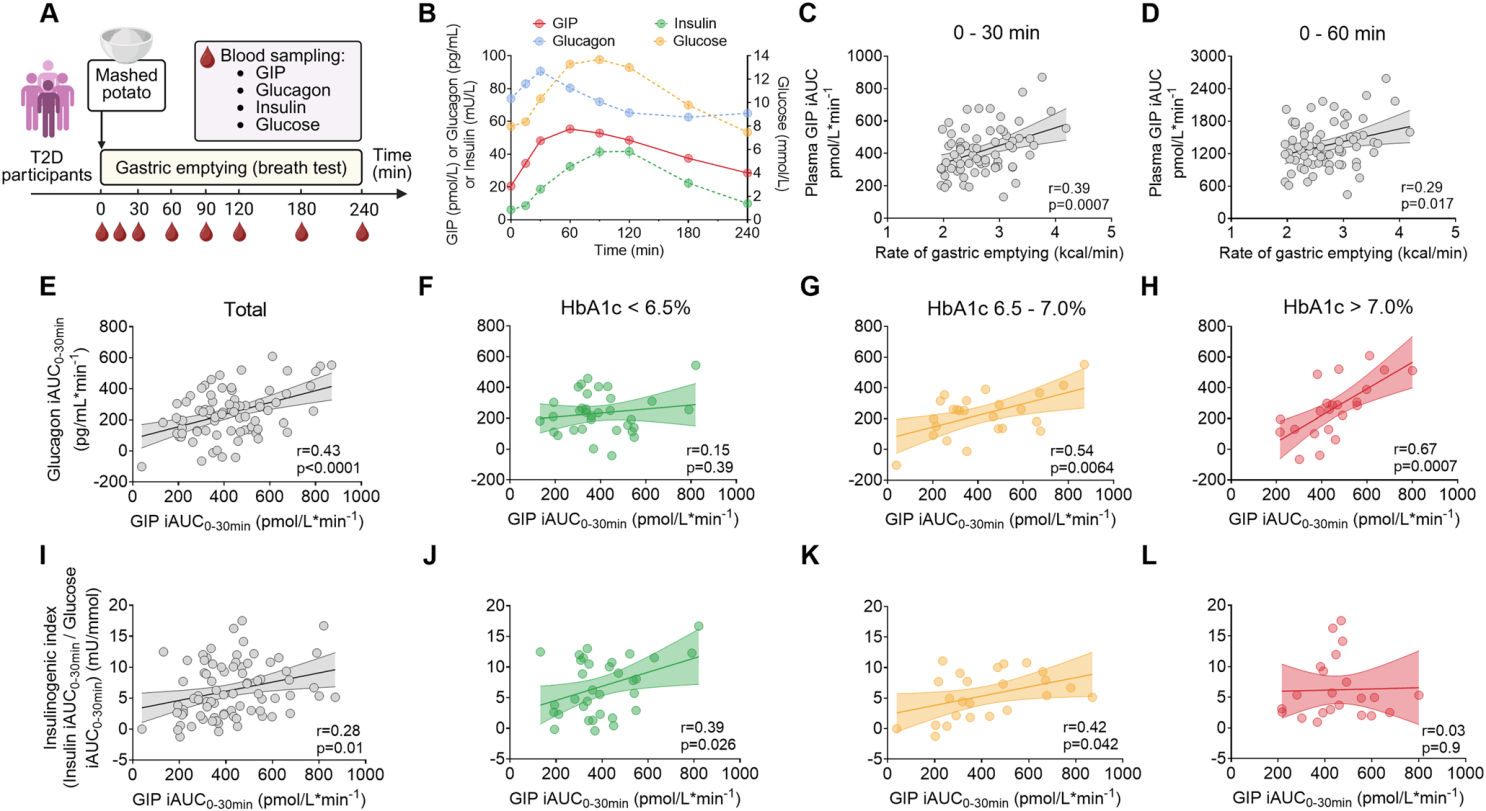
(A) Study protocol. (B) Blood glucose and hormonal responses to the mixed meal in T2D subjects (*n* = 79); data are means ± SEM. (C, D) Relationship between plasma total GIP iAUC during 0–30 min and 0–60 min and the rate of gastric emptying (*n* = 70). Relationship of postprandial glucagon (E, H) and insulin (I, L) with total GIP in the overall T2D cohort (*n* = 79) and subgroups with HbA1c levels < 6.5% (*n* = 33) (F, J), 6.5%–7.0% (*n* = 24) (G, K), and > 7.0% (*n* = 22) (H, L) respectively. Relationships between the plasma total GIP response and the rate of gastric emptying, as well as glucagon/insulin responses were assessed using Pearson correlation analysis after confirming normal distribution of data.

The rate of gastric emptying (available in 70 subjects) ranged from 1.96 to 4.19 kcal/min, with a mean rate of 2.70 ± 0.06 kcal/min. Following the meal, both blood glucose and plasma insulin levels increased gradually, peaking at ~90 min. Plasma glucagon concentrations increased within the first 30 min and then decreased to a nadir at 180 min. Plasma GIP concentrations increased during the first 30 min, peaking at ~60 min, followed by a slow decline (Figure 1B). Plasma GIP iAUC_0‐30min_ and iAUC_0‐60min_ were related directly to the rate of gastric emptying (*r* = 0.39, *p* < 0.001; *r* = 0.29, *p* = 0.017, respectively) (Figure 1C,D).

#### 3.1.2 Postprandial Glucagon, Insulin and GIP Secretion in Relation to Glycaemic Control in T2D

Plasma glucagon iAUC_0‐30min_ correlated positively with plasma GIP iAUC_0–30min_ (*r* = 0.43, *p* < 0.0001, *n* = 79) (Figure 1E). This relationship was strongest in the higher HbA1c subgroups (*r* = 0.67, *p* = 0.0007, *n* = 22), less in the intermediate HbA1c subgroup (*r* = 0.54, *p* = 0.0064, *n* = 24) and not evident in the lower HbA1c subgroup (*r* = 0.15, *p* = 0.39, *n* = 33) (Figure 1F–H). There was no difference in either sex distribution, age, BMI, metformin use, or HOMA‐IR between sub‐groups. The insulinogenic index correlated positively, but weakly, with plasma GIP iAUC_0‐30min_ (*r* = 0.28, *p* = 0.01, *n* = 79) (Figure 1I). This relationship was evident in the lower and intermediate HbA1c subgroups (*r* = 0.39, *p* = 0.026 and *r* = 0.42, *p* = 0.042, respectively), but not in the higher HbA1c subgroup (*r* = 0.03, *p* = 0.9) (Figure 1J–L).

### 3.2 Part 2

#### 3.2.1 Relationship Between Glucagon and GIP Responses to Intraduodenal Fat Infusion in T2D

**FIGURE 2:**
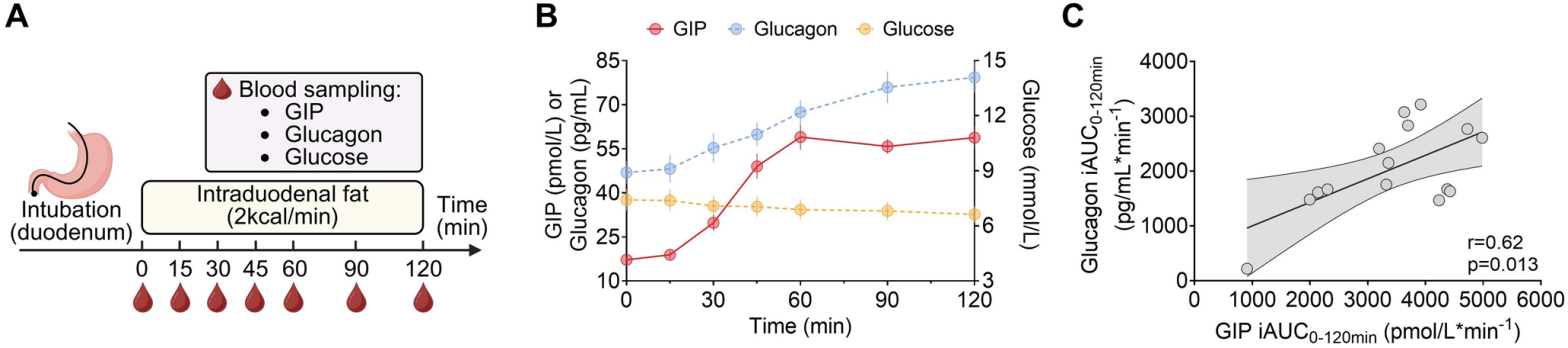
(A) Study protocol. (B) Blood glucose, total plasma GIP, and glucagon levels during intraduodenal fat infusion at 2 kcal/min in T2D; data are means ± SEM (*n* = 15). (C) Relationship between glucagon iAUC_0‐120min_ and total GIP iAUC_0‐120min_ in T2D (*n* = 15). The relationship between total GIP and glucagon responses was assessed using Pearson correlation analysis after confirming normal distribution of data.

Following intraduodenal fat infusion, plasma glucose concentrations remained relatively stable while both plasma glucagon and total GIP concentrations increased progressively over 120 min (Figure 2B). The magnitude of increase in plasma glucagon (i.e., plasma glucagon iAUC_0‐120min_) correlated positively with that of plasma GIP (*r* = 0.62, *p* = 0.013, Figure 2C).

### 3.3 Part 3

#### 3.3.1 Relationship Between Glucagon and GIP Responses to Intraduodenal Glucose Infusion in Health and T2D

**FIGURE 3:**
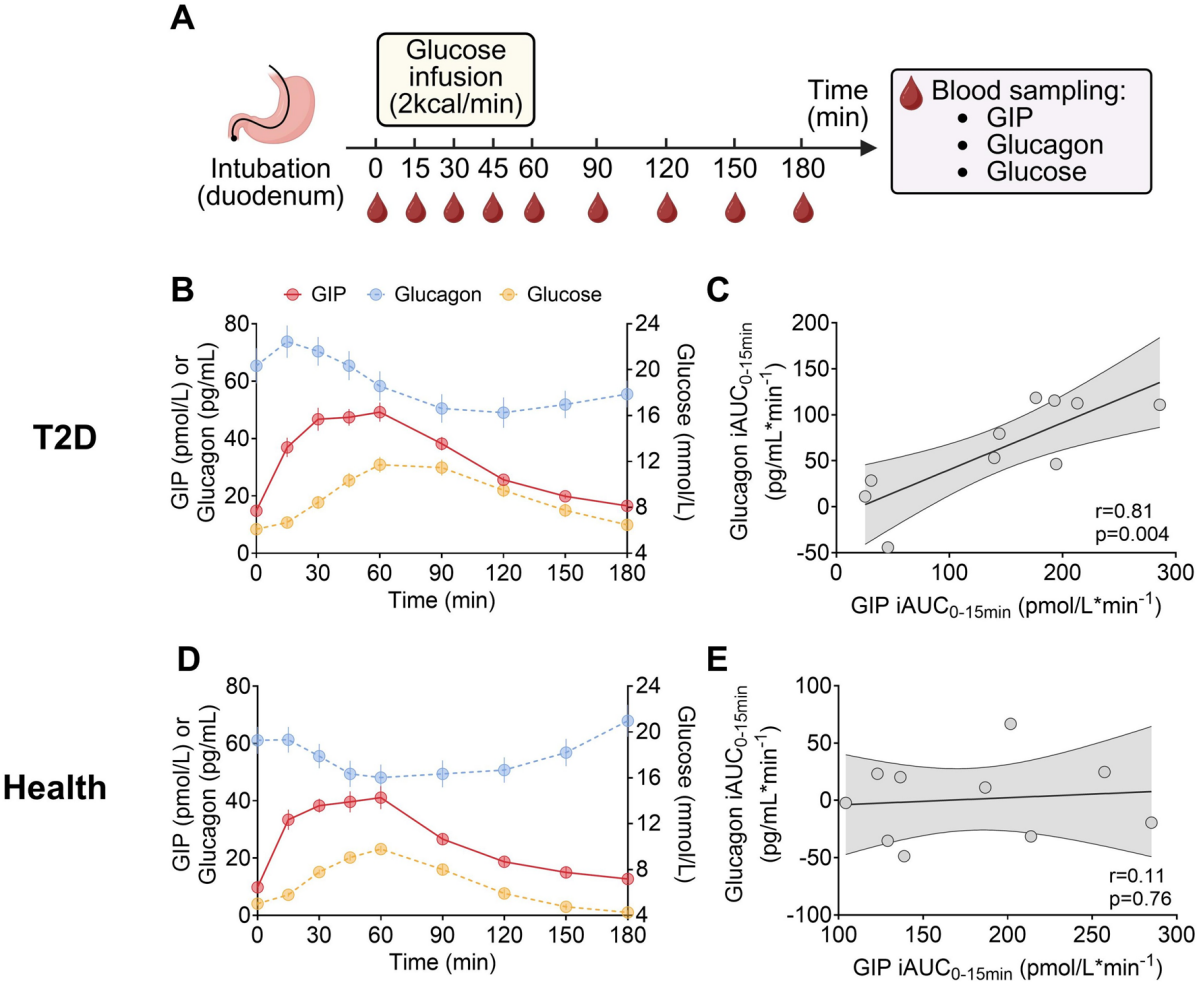
(A) Study protocol. (B, D) Blood glucose, total plasma GIP, and glucagon levels during intraduodenal glucose infusion at 2 kcal/min in (B) T2D subjects (*n* = 10) and (D) healthy subjects (*n* = 10); data are means ± SEM. (C, E) Relationships between glucagon iAUC_0‐15min_ and total GIP iAUC_0–15min_ in (C) T2D subjects and (E) healthy subjects. Relationships between total GIP and glucagon responses were assessed using Pearson correlation analysis after confirming normal distribution of data.

Blood glucose and plasma glucagon and GIP responses to intraduodenal glucose infusion in both healthy and T2D subjects are depicted in Figure 3B,D. In T2D subjects, there was a strong relationship between plasma glucagon iAUC_0‐15min_ and plasma GIP iAUC_0–15min_ (*r* = 0.81, *p* = 0.004, Figure 3C). In contrast, no relationship was evident in healthy subjects (*r* = 0.11, *p* = 0.76, Figure 3E).

## 4 Discussion

We demonstrate that the relationship between postprandial GIP secretion and glucagon/insulin responses in T2D is strongly modulated by antecedent chronic glycaemic control. Specifically, we showed that: (i) in a group of T2D participants with relatively good glycaemic control, during a mixed‐meal test, the ‘early’ postprandial (0–30 min) glucagon response correlated positively with GIP secretion, with the strength of this association increasing in subgroups with relatively higher HbA1c levels, whereas the insulinogenic index—a marker of early‐phase β‐cell response—correlated positively with GIP only in those with HbA1c ≤ 7.0%; (ii) during intraduodenal fat infusion, the plasma glucagon response over 120 min correlated strongly with that of GIP in T2D and (iii) following intraduodenal glucose infusion, early increments in plasma glucagon and GIP were closely related in T2D, but not in healthy, individuals. Together, these observations indicate a chronic glycaemia‐dependent shift in GIP action in T2D, characterised by attenuation of its insulinotropic and relative amplification of its glucagonotropic, effect as glycaemic control deteriorates.

Following meal ingestion, glucagon concentrations increased rapidly during the early postprandial period before declining despite continued increases in GIP. This temporal pattern likely reflects the dynamic interplay between multiple regulators of α‐cell secretion. In particular, rising plasma glucose, insulin and GLP‐1 concentrations later in the postprandial period are known to suppress glucagon secretion. Accordingly, our analyses focused on the early postprandial phase, when nutrient‐stimulated GIP secretion is most likely to influence glucagon release before these inhibitory factors become dominant.

Consistent with the established dual action of GIP on pancreatic β‐ and α‐cells, we observed positive associations between ‘early’ GIP responses and the release of both insulin and glucagon in individuals with well‐controlled T2D. However, with worsening glycaemia, GIP‐insulin coupling weakened, consistent with evidence that chronic glucotoxicity impairs β‐cell GIP responsiveness, a defect that is partially reversible with glucose‐lowering therapy [10, 18]. In contrast, the progressive strengthening of GIP‐glucagon coupling with increasing HbA1c suggests a relative dominance of glucagonotropic action in poorer glycaemic states.

Given that T2D is characterised by both impaired insulin secretion and inappropriate glucagon release, the glycaemia‐related shift in GIP‐islet hormone coupling may contribute to the pathophysiology of T2D. Supporting this concept, acute exogenous GIP administration was shown to augment glucagon secretion and worsen postprandial glycaemia in suboptimally controlled T2D [11]. However, glucagon also exhibits pleiotropic actions on energy expenditure, lipid metabolism and body weight [19, 20], raising the possibility that enhanced GIP‐glucagon coupling in individuals with poorer glycaemic control may represent a compensatory response to metabolic stress. This interpretation aligns with the metabolic benefits of dual GLP‐1/GIP and emerging triple GLP‐1/GIP/glucagon receptor agonists.

The glycaemia‐dependent shift in GIP‐islet hormone relationships may reflect glucotoxicity‐induced internalisation or downregulation of β‐cell GIP receptors, leading to reduced insulinotropic responsiveness [21, 22]. Diminished β‐cell activity may, in turn, reduce paracrine inhibition of α‐cells [23], thereby unmasking or enhancing GIP‐mediated glucagon secretion. Given the pivotal role of gastric emptying in nutrient delivery and incretin hormone release [24] and prior links between ‘early’ glucagon responses to oral glucose and mixed meals and gastric emptying in T2D [12], we examined the relationship between glucagon and GIP secretion during intraduodenal nutrient delivery. In the absence of changes in plasma glucose and insulin, glucagon iAUC_0‐120min_ correlated strongly with that of GIP following intraduodenal fat infusion in T2D participants. Although fatty acids can stimulate glucagon secretion [25], the similarly strong early correlation between glucagon and GIP during intraduodenal glucose infusion in T2D argues against a dominant lipid‐mediated effect. In healthy individuals, robust early insulin responses may have obscured GIP‐mediated glucagon effects. Collectively, these findings support a close and glucose‐state‐dependent link between GIP and glucagon secretion in T2D independent of gastric emptying.

Our findings suggest that the balance between the insulinotropic and glucagonotropic effects of endogenous GIP signalling may vary according to the prevailing glycaemic milieu. This raises the possibility that GIP receptor agonism could confer greater glucose‐lowering efficacy in individuals with better‐controlled T2D. However, endogenous GIP secretion/signalling is not directly comparable to pharmacological GIP receptor activation, particularly because long‐acting GLP‐1/GIP co‐agonists provide sustained receptor stimulation in both fasting and postprandial states. Accordingly, the translational implications of our findings should be considered hypothesis‐generating, and prospective studies are needed to determine whether similar glycaemia‐dependent effects occur with pharmacological GIP receptor agonism.

Several limitations warrant consideration. First, the observational nature of the present study precludes causal inference. Accordingly, the relationships observed between endogenous GIP secretion and glucagon responses should be interpreted as associations rather than direct physiological effects. Further studies will be required to determine whether these relationships reflect causal mechanisms linking GIP signalling to α‐cell function in T2D. Second, the experimental settings of Parts 1 and 3 have inherent limitations to evaluate the relationship between GIP and islet hormones over the entire study period due to the concurrent changes in multiple factors known to influence glucagon secretion. Future studies employing more controlled experimental designs, such as glycaemic clamps, may help to minimise these confounding factors and allow more precise assessments of the relationship between GIP and islet hormone responses. Third, the sample sizes in some experiments were modest and all T2D participants had HbA1c < 8%; accordingly, the relationships observed between GIP and glucagon/insulin in individuals with poorer glycaemic control remain to be determined. Fourth, healthy control data were not available for the mixed meal and intraduodenal fat infusion protocols, as these datasets were derived from previously published studies designed specifically to examine metabolic responses in T2D. However, in the intraduodenal glucose infusion study, both healthy individuals and participants with T2D were studied under identical experimental conditions, allowing direct comparisons of hormonal responses between these groups.

In conclusion, we demonstrate a progressive, glycaemia‐dependent shift in the relationship between endogenous GIP and glucagon responses in T2D, independent of gastric emptying and macronutrient type. As chronic glycaemic control worsens, the association between GIP and insulin secretion weakens, whereas the relationship between GIP and glucagon responses strengthens. These findings highlight the complex role of GIP in islet hormone regulation and suggest that the metabolic effects of GIP signalling may be dependent on the prevailing glycaemic status.

## Author Contributions

Z.W. was involved in data collection and interpretation, statistical analysis, and writing of the manuscript. W.H. and R.S.R. were involved in data analysis and reviewing of the manuscript. M.H., K.L.J. and C.K.R. were involved in conception and design of the study, data interpretation, and reviewing of the manuscript. C.X. was involved in the design of the study, data collection and interpretation, statistical analysis and reviewing of the manuscript. T.W. was involved in the conception and design of the study, data interpretation, statistical analysis and drafting of the manuscript and is the guarantor of this work. All authors have approved this final version of the manuscript.

## Funding

The studies were supported by the NHMRC project grant (ID: APP10066835) and Novartis. W.H. is supported by Australian Diabetes Society Skip Martin Fellowship. C.K.R. is supported by the Michell Bequest Foundation. C.X. is supported by Central Adelaide Local Health Network Florey Fellowship. T.W. is supported by the Australian Medical Research Future Fund (MRFCDDM000009).

## Conflicts of Interest

C.K.R. has received research funding from Glyscend Theraputics. T.W. has received research funding from DSM‐Firmenich. The other authors declare no conflicts of interest.

## Data Availability

Study data are available from the corresponding authors upon reasonable request.
